# Supplementary material for: From sequence to enzyme mechanism using multi-label machine learning
Source: BMC Bioinformatics. 2014 May 19;15:150. doi: 10.1186/1471-2105-15-150 (PMC4229970; doi:10.1186/1471-2105-15-150)
Supplement: Additional file 2 — Java code of ml2db. Additional file ml2db_code.tar.gz contains the Java source code to run the multi-label machine learning experiments and save the results to database. The code’s Javadoc is included. [file 1471-2105-15-150-S2.zip › additional file 2/ml2db/utils/doc/uk/ac/standrews/utils/main/webutils/simpledomparser/XmlSearcher.html]

XmlSearcher


JavaScript is disabled on your browser.


- Overview
- Package
- Class
- Use
- Tree
- Deprecated
- Index
- Help

- Prev Class
- Next Class

- Frames
- No Frames

- All Classes

- Summary:
- Nested |
- Field |
- Constr |
- Method

- Detail:
- Field |
- Constr |
- Method


uk.ac.standrews.utils.main.webutils.simpledomparser

## Class XmlSearcher

- java.lang.Object
- - uk.ac.standrews.utils.main.webutils.simpledomparser.XmlSearcher

- ---

    

  ```
  public class XmlSearcher
  extends java.lang.Object
  ```

  Executes a search over a SimpleElement (an XML tree)

  Version:
  :   27 Feb 2009

  Author:
  :   Luna De Ferrari luna.deferrari-at-ed.ac.uk

- - ### Field Summary

    Fields

    | Modifier and Type | Field and Description |
    | --- | --- |
    | `static boolean` | `m_debug` |
  - ### Constructor Summary

    Constructors

    Constructor and Description

    `XmlSearcher()`
  - ### Method Summary

    Methods

    | Modifier and Type | Method and Description |
    | --- | --- |
    | `static XmlNode` | `depthFirstSearch(XmlNode xmlTree, java.lang.String searchString, java.lang.String attributeName, java.io.File log)` Searches this tree in a depth first mode and returns the first node whose tag, text or attribute value matches the given string. |
    | `static XmlNode` | `depthFirstSearch(XmlNode xmlTree, java.lang.String searchString, java.lang.String attributeName, java.io.File logFile, int emergencyBrake, int maxNumberOfChildren)` Searches this tree in a depth first mode and returns the first node whose tag, text or attribute value matches the given string. |
    | `static XmlNode` | `depthFirstSearchOfParent(XmlNode tree, java.lang.String desiredTag, java.lang.String attributeName)` |
    | `static XmlNode` | `depthFirstSearchOfParent(XmlNode xmlTree, java.lang.String searchString, java.lang.String attributeName, java.io.File logFile, int emergencyBrake, int maxNumberOfChildren)` TODO duplicated code! depthFirstSearch ~ depthFirstSearchOfParent Searches this tree in a depth first mode and returns the \*parent\* of the first node whose tag, text or attribute value matches the given string. |
    | `static boolean` | `evaluateCondition(XmlNode element, java.lang.String wantedString)` Check whether the desired string is either a tag or text for this element. |
    | `static boolean` | `evaluateCondition(XmlNode element, java.lang.String wantedString, java.lang.String attributeName)` Check whether the desired string is either a tag, text or an attribute \*value\* for this element. |
    | `static java.util.LinkedList<XmlNode>` | `getInternalBlocks(java.lang.String externalBlockTag, XmlNode xmlTree, java.io.File log)` Gets the children of a given xml tag |
    | `static java.lang.String` | `getTextFromChildElement(XmlNode xmlTree, java.lang.String searchString, java.lang.String attributeName, java.io.File log)` Checks if child exists and returns its text |

    - ### Methods inherited from class java.lang.Object

      `equals, getClass, hashCode, notify, notifyAll, toString, wait, wait, wait`

- - ### Field Detail


    - #### m\_debug

      ```
      public static boolean m_debug
      ```
  - ### Constructor Detail


    - #### XmlSearcher

      ```
      public XmlSearcher()
      ```
  - ### Method Detail


    - #### depthFirstSearch

      ```
      public static XmlNode depthFirstSearch(XmlNode xmlTree,
                             java.lang.String searchString,
                             java.lang.String attributeName,
                             java.io.File log)
      ```

      Searches this tree in a depth first mode and returns the first node whose
      tag, text or attribute value matches the given string. If not found,
      stops after a default number of actions (usually 5000, but check the java
      code) or a certain number of children (usually 5000).

      Parameters:
      :   `searchString` - the text to be found
      :   `attributeName` - the attribute name (if available, but it can be null)

      Returns:
      :   the first element whose tag/text/attribute value matches the
          wanted text


    - #### depthFirstSearch

      ```
      public static XmlNode depthFirstSearch(XmlNode xmlTree,
                             java.lang.String searchString,
                             java.lang.String attributeName,
                             java.io.File logFile,
                             int emergencyBrake,
                             int maxNumberOfChildren)
      ```

      Searches this tree in a depth first mode and returns the first node whose
      tag, text or attribute value matches the given string.

      Parameters:
      :   `xmlTree` - the tree to be searched
      :   `searchString` - the text to be found in a node
      :   `attributeName` - the attribute name (if available, but it can be null)
      :   `logFile` - a log file (if a file output for the log is desired, or null
          to obtain a console printout)
      :   `emergencyBrake` - the number of tags to check before abandoning the search and
          returning null
      :   `maxNumberOfChildren` - if a tag has more than this number of children, the children
          will not be considered in the search

      Returns:
      :   the first element whose tag/text/attribute value matches the
          wanted text


    - #### depthFirstSearchOfParent

      ```
      public static XmlNode depthFirstSearchOfParent(XmlNode tree,
                                     java.lang.String desiredTag,
                                     java.lang.String attributeName)
      ```


    - #### depthFirstSearchOfParent

      ```
      public static XmlNode depthFirstSearchOfParent(XmlNode xmlTree,
                                     java.lang.String searchString,
                                     java.lang.String attributeName,
                                     java.io.File logFile,
                                     int emergencyBrake,
                                     int maxNumberOfChildren)
      ```

      TODO duplicated code! depthFirstSearch ~ depthFirstSearchOfParent
      Searches this tree in a depth first mode and returns the \*parent\* of the
      first node whose tag, text or attribute value matches the given string.
      Having the parent allows, for example, to delete the child node.

      Parameters:
      :   `xmlTree` - the tree to be searched
      :   `searchString` - the text to be found in a node
      :   `attributeName` - the attribute name (if available, but it can be null)
      :   `logFile` - a log file (if a file output for the log is desired, or null
          to obtain a console printout)
      :   `emergencyBrake` - the number of tags to check before abandoning the search and
          returning null
      :   `maxNumberOfChildren` - if a tag has more than this number of children, the children
          will not be considered in the search

      Returns:
      :   the \*parent\* of the first node whose tag/text/attribute value
          matches the wanted text


    - #### evaluateCondition

      ```
      public static boolean evaluateCondition(XmlNode element,
                              java.lang.String wantedString)
      ```

      Check whether the desired string is either a tag or text for this
      element.

      Parameters:
      :   `element` -
      :   `wantedString` -

      Returns:
      :   the element if it matches, null otherwise


    - #### evaluateCondition

      ```
      public static boolean evaluateCondition(XmlNode element,
                              java.lang.String wantedString,
                              java.lang.String attributeName)
      ```

      Check whether the desired string is either a tag, text or an attribute
      \*value\* for this element.

      Parameters:
      :   `element` -
      :   `wantedString` -
      :   `attributeName` - if the attribute name is specified, the method checks the
          wanted string against the attribute value

      Returns:
      :   the element if it matches, null otherwise


    - #### getInternalBlocks

      ```
      public static java.util.LinkedList<XmlNode> getInternalBlocks(java.lang.String externalBlockTag,
                                                    XmlNode xmlTree,
                                                    java.io.File log)
      ```

      Gets the children of a given xml tag

      Parameters:
      :   `externalBlockTag` - the tag that identifies the block

      Returns:
      :   the children blocks insied the given xml tag


    - #### getTextFromChildElement

      ```
      public static java.lang.String getTextFromChildElement(XmlNode xmlTree,
                                             java.lang.String searchString,
                                             java.lang.String attributeName,
                                             java.io.File log)
      ```

      Checks if child exists and returns its text

      Parameters:
      :   `searchString` - the tag or content string of the desired child
      :   `attributeName` - the attribute name (if available, but it can be null if a
          search string is passed)

      Returns:
      :   the text of the child or null if a child containing the search
          string could not be found


- Overview
- Package
- Class
- Use
- Tree
- Deprecated
- Index
- Help

- Prev Class
- Next Class

- Frames
- No Frames

- All Classes

- Summary:
- Nested |
- Field |
- Constr |
- Method

- Detail:
- Field |
- Constr |
- Method
